# Attempts to use breeding approaches in *Aedes aegypti* to create lines with distinct and stable relative *Wolbachia* densities

**DOI:** 10.1038/s41437-022-00553-x

**Published:** 2022-07-22

**Authors:** A. J. Mejia, L. Jimenez, H. L. C. Dutra, R. Perera, E. A. McGraw

**Affiliations:** 1 Center for Infectious Disease Dynamics & Department of Entomology, The Pennsylvania State University, University Park, PA 16802 USA; 2 School of Life Sciences, Monash University, Clayton, Vic 3800 Australia; 3 Center for Infectious Disease Dynamics & Biology, The Pennsylvania State University, University Park, PA 16802 USA; 4 Center for Vector-borne Infectious Diseases and Center for Metabolism of Infectious Diseases, Department of Microbiology, Immunology and Pathology, Colorado State University, Fort Collins, CO USA

**Keywords:** Evolutionary biology, Experimental evolution

## Abstract

*Wolbachia* is an insect endosymbiont being used for biological control in the mosquito *Aedes aegypti* because it causes cytoplasmic incompatibility (CI) and limits viral replication of dengue, chikungunya, and Zika viruses. While the genetic mechanism of pathogen blocking (PB) is not fully understood, the strength of both CI and PB are positively correlated with *Wolbachia* densities in the host. *Wolbachia* densities are determined by a combination of *Wolbachia* strain and insect genotype, as well as interactions with the environment. We employed both artificial selection and inbreeding with the goal of creating lines of *Ae. aegypti* with heritable and distinct *Wolbachia* densities so that we might better dissect the mechanism underlying PB. We were unable to shift the mean relative *Wolbachia* density in *Ae. aegypti* lines by either strategy, with relative densities instead tending to cycle over a narrow range. In lieu of this, we used *Wolbachia* densities in mosquito legs as predictors of relative densities in the remaining individual’s carcass. Because we worked with outbred mosquitoes, our findings indicate either a lack of genetic variation in the mosquito for controlling relative density, natural selection against extreme densities, or a predominance of environmental factors affecting densities. Our study reveals that there are moderating forces acting on relative *Wolbachia* densities that may help to stabilize density phenotypes post field release. We also show a means to accurately bin vector carcasses into high and low categories for non-DNA omics-based studies of *Wolbachia*-mediated traits.

## Introduction

*Aedes aegypti’s* geographic range is expanding globally (Kraemer et al. 2019). This mosquito transmits human disease-causing viruses including dengue (DENV), chikungunya (CHIKV), Yellow Fever (YFV), and Zika (ZIKV) (Souza-Neto et al. 2019). There are currently no effective vaccines or antiviral drugs for these arboviruses (Merle et al. 2018), except for YFV. Instead, we rely on vector control to suppress arboviral populations. *Wolbachia pipientis* is a bacterial endosymbiont found in ~40% of insect species (Zug and Hammerstein 2015). *Wolbachia* induces two traits in mosquitoes that are the basis of its utility in vector control (Flores and O’Neill 2018), cytoplasmic incompatibility (CI) and *Wolbachia*-Mediated Pathogen Blocking (PB). CI manifests as embryonic death due to modifications to the sperm made by *Wolbachia* (Werren 1997). CI occurs when *Wolbachia*-free females mate with infected males, whereas all remaining crosses, result in viable offspring. CI results in females with *Wolbachia* having greater reproductive success in mixed populations. This advantage, combined with *Wolbachia’s* vertical inheritance, causes the symbiont to spread quickly through populations (Werren et al. 2008). *Wolbachia* has also been found to limit viral replication in various insects through a trait known as PB (Bian et al. 2010; Dutra et al. 2016; Moreira et al. 2009). PB was first discovered in *Drosophila*, when insects infected with viruses exhibited longer lifespans if they also were infected with *Wolbachia* (Hedges et al. 2008). It was determined that *Wolbachia* protects flies by reducing viral loads of the coinfecting virus (Teixeira et al. 2008). This same viral blocking effect has been seen in *Ae. aegypti* for DENV, CHIKV, YFV, and ZIKV (Dutra et al. 2016; van den Hurk et al. 2012; Moreira et al. 2009), after artificially, but stably infecting this species that is naturally *Wolbachia*-free, with *Wolbachia* from donor species (McMeniman et al. 2009; Walker et al. 2011).

This combination of CI and PB has created the ideal biological control agent against arboviruses. By spreading *Wolbachia* infection into wild *Ae. aegypti* populations through field release of *Ae. aegypti* females and the action of CI, it is possible to replace the local population with one that is largely resistant to virus transmission (Flores and O’Neill 2018). Currently, two main *Wolbachia* strains are being released globally, *w*Mel and *w*AlbB (Hoffmann et al. 2011; Nazni et al. 2019) that are derived from *Drosophila melanogaster* (Walker et al. 2011) and *Aedes albopictus* (Xi et al. 2005), respectively. Both reduce DENV replication (Nazni et al. 2019; Walker et al. 2011) and have effects on host fitness as measured in the lab (Axford et al. 2016; Hoffmann et al. 2014). The fitness effects have not hindered releases in Australia (Ryan et al. 2020), Malaysia (Nazni et al. 2019), and Indonesia (Utarini et al. 2021), but may be causing issues in Brazil (Pinto et al. 2021) and Vietnam (Hien et al. 2022). The *w*AlbB strain is more tolerant to cyclical heat stress than *w*Mel, suggesting that *w*AlbB may have higher success in environments where mosquitoes experience heat stress (Ross et al. 2017).

The mechanism of *Wolbachia*-mediated pathogen blocking in mosquitoes is still not fully understood. From a range of studies, it is clear that the trait is likely multifaceted (Lindsey et al. 2018). Without the ability to genetically modify *Wolbachia*, much of the focus has been on identifying the effects of viral blocking in the mosquito. Processes in the mosquito such as immunity, nutrient competition, RNA translation and replication, and cellular stress are affected by *Wolbachia* and may assist with viral blocking (Ford et al. 2020; Geoghegan et al. 2017; Moreira et al. 2009; Rainey et al. 2016; Rancès et al. 2012; White et al. 2017). Multiple studies have shown that *Wolbachia* upregulates immune gene expression in *Ae. aegypti* (Moreira et al. 2009; Rancès et al. 2012). This priming of the immune system in *Ae. aegypti* may increase its basal immune protection, allowing the mosquito to have greater control over viruses it subsequently encounters during blood feeding. Another suggested basis of blocking is competition for cholesterol. Both dengue virus and *Wolbachia* depend on cholesterol metabolism for survival and replication (Geoghegan et al. 2017; Heaton et al. 2010). Viral RNA translation and/or replication appears to be limited when *Wolbachia* is present (Rainey et al. 2016), possibly through alteration of the host’s endoplasmic reticulum and golgi complex that viruses use to replicate (White et al. 2017). This change may make the endoplasmic reticulum and golgi complex unsuitable for viruses (Lindsey et al. 2018). Last, *Wolbachia* induces host cellular stress represented by increased levels of reactive oxygen species (Pan et al. 2012). Reactive oxygen species activate signaling pathways such as the extracellular signal-regulated kinase pathway (Thannickal and Fanburg 2000), which has been demonstrated to increase viral protection in mosquito cells (Xu et al. 2013).

Regardless of the specific mechanism, blocking strength has been shown to correlate with relative *Wolbachia* densities in whole insects (Amuzu and McGraw 2016; Chouin-Carneiro et al. 2020; Chrostek et al. 2013; Iturbe-Ormaetxe et al. 2011; Joubert et al. 2016; Rainey et al. 2016). *Wolbachia* density could therefore be used to help study the basis of pathogen blocking, and indeed other *Wolbachia*-associated traits, if we could generate high and low-density lines in mosquitoes. Previous studies have compared closely related (Woolfit et al. 2013) *Wolbachia* strains, known to vary in their densities such as *w*Mel as compared to *w*MelPop (Walker et al. 2011) and *w*MelPop-CLA (Joubert et al. 2016), but such approaches are confounded by other genetic differences between the strains and their associated phenotypes including virulence for both the latter two strains. Similarly, comparing the same *Wolbachia* strain across different vector strains or species where relative densities may vary, includes the confounding effects of other genetic differences between vectors, unrelated to control of relative densities (Ikeda et al. 2003; McGraw et al. 2002). For example, *Ae. albopictus* is naturally co-infected with two *Wolbachia* strains, *w*AlbA, and *w*AlbB, that have a relative abundance of 1:10 in the native host (Dutton and Sinkins 2004). When transinfected into *Ae. aegypti* the relationship reverses however, with *w*AlbA exhibiting a greater relative density than *w*AlbB (Ant et al. 2018). Our goal in this study was to create genetically similar, independent lines of *Ae. aegypti* with stable and distinct differences in their *Wolbachia* densities originating from a single original strain of *Wolbachia*. Because genetic variation is likely very low in *Wolbachia* populations due to the bottlenecking at the point of creation of the original transfected *Ae. aegypti* line (Fraser et al. 2020) and at each generation through the packaging of symbionts into the embryo (Newton et al. 2015; Zug and Hammerstein 2015), we were reliant on any standing genetic variation in the vector that may affect *Wolbachia* density (Kondo et al. 2005; Mouton et al. 2007) to assist with line creation. Here we utilized multiple approaches involving artificial selection, inbreeding, and tissue-based correlation to create predictably high and low-density lines or individuals that could be used further for trait decomposition.

## Methods

### *Ae. aegypti* rearing

Artificial selection was carried out on *Ae. aegypti* infected with the *w*Mel strain of *Wolbachia* (Hoffmann et al. 2011; Walker et al. 2011) in mosquitoes recently collected (within 3 generations of the field) from Cairns, Australia. Hundreds of eggs were collected from ovitraps placed at 6 sites across greater Cairns as per previous (Frentiu et al. 2014). Fourth instar larvae were identified to species based on morphological characters. A founding population of ~500 mosquitoes was created by pooling larvae equally across the 6 sites. The tissue correlation and inbreeding experiments were carried out several years later using *w*AlbB infected *Ae. aegypti* (Xi et al. 2005) obtained from Zhiyong Xi (Michigan State) as the *w*Mel strain was no longer available due to MTA restrictions. As this line had been bred in the lab for over a year when it was obtained, we backcrossed *w*AlbB into a wildtype line from Monterrey, Mexico for 3 generations to increase genetic diversity. Each cross involved ~200 females and males from each line. The wild Monterrey line provided by Matthew Thomas (Penn State), had been in the laboratory <3 generations and was initially generated by pooling thousands of eggs collected from ovitraps placed across locations in Monterrey. For all experiments, mosquito eggs were hatched in 40 × 30 × 8 cm plastic trays with 3 liters of autoclaved reverse-osmosis water and fed Tetramin fish food (Melle, Germany) *ad libitum*. Larvae were maintained at a density of ~250 per tray. Populations of ~300 adult mosquitoes were housed in 18 × 18” square breeding cages (BioQuip). Dental wicks were used to provide access to 10% sucrose. Mosquitoes were fed human blood (BioIVT) warmed to 37 °C using an artificial feeder (Hemotek) at 9–11 days of age to collect eggs and maintain the colonies.

### Artificial selection experiment

We employed an artificial selection regime to create mosquito lines with increased relative *Wolbachia* densities in the whole body of the mosquito with the goal of studying the basis of DENV blocking. A total of 480 blood-fed (human volunteer, ethics permit number CF11/0766-2011000387) *w*Mel *Ae. aegypti* females were placed individually in 70 mL plastic cups (Sarstedt). Eggs were collected using moist filter paper. Females that laid eggs were then collected for DNA extraction and *Wolbachia* density measurement (below). We ran three selection lines (S1-3) and three control lines (C1-3) in parallel. We created the selection lines by pooling 100 eggs from each of 3 females with the highest *Wolbachia* density, and the control lines by pooling the offspring from three females randomly selected with respect to titre. We created each subsequent generation in the same way, by assaying 80 randomly selected females (post isofemaling and egg collection) for *Wolbachia* density and choosing the three mosquitoes with the highest titre (S1-3) or three mosquitoes (C1-3) chosen by a random number generator to each contribute 100 eggs. To obtain the eggs from said females above, all lines were blood-fed 6–8 days post-eclosion by a human volunteer in large populations. Then 12–16 h after blood-feeding, 80 blood fed females were selected at random and placed in individual 70 mL cups (Sarstedt) as isofemales. They were provided with 10% sucrose solution and moist filter paper for egg collection, and cups were checked for eggs after three days. Dead females and those that laid <10 eggs were discarded. Eighty females were isolated for eventual qPCR for each of the three cages and assessed for relative *Wolbachia* densities. Females were ranked by relative *Wolbachia* density, and eggs from the highest density females were pooled to seed the next generation for the individual line/cage. The number of females used to seed the next generation was consistent across all three cages. A similar process was used for the three control cages, except females and their offspring were randomly chosen to seed the subsequent generation. The selection regime was carried out for a total of 4 sequential generations (continuous selection).

### Isofemale line experiment

We also employed an isofemale line approach to see if we could generate lines with distinct and predictable relative *Wolbachia* densities (high vs. low) in *w*AlbB *Ae. aegypti*. To create isofemale lines, we subsequently reared the offspring collected from 41 single pair crosses separately as small, closed populations (30–50 individuals) for 8–9 generations. Given issues with fitness for many of the lines, only 8 lines of the original 41 survived. The original 41 P_1_ females were dissected for ovaries and the remaining carcass at 15–17 days of age, or ~6 days post feed and egg-laying for *Wolbachia* density determination. We fed the eight remaining isofemale lines at each generation and then collected their eggs in 70 ml oviposition cups containing moist filter paper. Egg papers were hatched independently for each line, and adults were reared in cages (as above). After 8–9 generations of breeding, we carried out individual tissue dissection and relative *Wolbachia* density estimates for 24–25 females per line as in the parental generation. After *Wolbachia* quantification, we focused our subsequent rearing efforts on four lines, the two highest and two lowest lines with respect to relative *Wolbachia* densities in the carcass. Finally, at generations 11–13, we dissected ovary and carcass tissues again for the subset of lines for comparison of relative *Wolbachia* densities to the P_1_ and F_8-9_ generations.

### Tissue correlation

After failing to select for high and low relative *Wolbachia* densities across generations by selection or isofemale line creation, we sought to determine whether we could predict *Wolbachia* density in the carcass or specific tissues based on first screening the mosquito legs. In brief, we dissected and pooled all 6 legs from individual *w*AlbB *Ae. aegypti* and either kept the remainder of the body (minus gonads) or specific tissues including the midgut and the salivary glands for subsequent relative *Wolbachia* density determination as per below. We initially attempted to correlate relative densities from a single leg but found that these estimates lacked sensitivity and repeatability compared to pooling all 6. Gonads were excluded given their extremely high relative *Wolbachia* densities that may swamp estimates in the much less dense somatic tissues responsible for the expression of PB. All dissections were carried out at 15–17 days of age, or ~6 days post feed. Each experiment utilized 25–40 individuals.

### Dissections and DNA extraction

In our artificial selection experiment, females were frozen and placed in 96-well plate (VQR Lab Advantage) with 50 μl of extraction buffer (10 mM Tris buffer, 1 mM EDTA, 50 mM NaCl, and proteinase K) and a 2-mm glass bead. Plates were homogenized with a MiniBeadbeater-96 (Bio Spec) for 90 s, centrifuged at 3220 × *g* for 3 min and then incubated at 58 °C for 30 s and at 96 °C for 5 min. In our isofemale line and tissue correlation experiments, females were cold-anesthetized and dissected in 1x phosphate-buffered saline (PBS). Tissues were collected and placed in a 2 ml tube with 50 μl of PBS and a 3-mm glass bead. Dissected tissues were stored at −80 °C until processing. To extract DNA, tubes were filled with 50 μl of extraction buffer. Samples were homogenized with a bead ruptor (OMNI International) for 90 s, centrifuged at 2000 × *g* for 2 min and then incubated at 56 °C for 5 min and at 98 °C for 5 min. A final centrifugation step was performed at 2000 × *g* for 2 min to pellet any remaining mosquito tissue. Samples were diluted 1:10 using DNAse/RNAse free water prior to quantification.

### *Wolbachia* quantification

Relative *Wolbachia* density was quantified through qPCR using Livak’s method (Livak and Schmittgen 2001). In brief, estimates of gene copy number are obtained for a single copy *Wolbachia* gene and host gene, that exhibit similar replication efficiencies in PCR. The ratio of the two, therefore represents an average estimate of *Wolbachia* per host cell in the tissue or whole animal being assessed. In the artificial selection experiment, we used the primers for the single copy ankyrin repeat containing gene *Wolbachia tm513* (previously WD513) (Woolfit et al. 2013) and the mosquito ribosomal subunit protein S17 gene (*rps17*). Primers: TM513_F (5’-CAAATTGCTCTTGTCCTGTGG) and TM513_R (5’-GGGTGTTAAGCAGAGTTACGG), as well as mosquito primers *RPS17*_F (5’-TCCGTGGTATCTCCATCAAGCT) and *RPS17*_R (5’-CACTTCCGGCACGTAGTTGTC) (Ford et al. 2019). We also used a fluorescent probe for TM513 and *RPS17*. Probes: TM513 probe (5’-Lc640-TGAAATGGAAAAATTGGCGAGGTGTAGG-Iowablack) and *RPS17* probe (5’-FAM-CAGGAGGAGGAACGTGAGCGCAG-BHQ1) via qPCR on a LightCycler 480 (Roche), using the equation $\frac{{2^{ - TM513}}}{{2^{ - RPS17}}}$. The artificial selection experiment consisted of a 10 μL final volume reaction, each containing 5 μL of LightCycler 480 Mastermix (Roche), 0.25 μL of each *RPS17* primer, 0.1 μL of *RPS17* probe, 0.3 μL of each TM513 primers, 0.3 μL of TM513 probe, 2.5 μL of nuclease-free water and 1uL of template DNA. For the isofemale line and tissue correlation experiments we also used the primer RPS17, but instead of TM513, we used previously published primers specific for *w*AlbB in an *ankyrin repeat domain* gene (Axford et al. 2016); Primer: *w*AlbB_F (5’-CCTTACCTCCTGCACAACAA) and *w*AlbB_R (5’-GGATTGTCCAGTGGCCTTA). We also switched to using a SYBR green approach. All qPCR was carried out on a LightCycler 480 (Roche), using the equation $\frac{{2^{ - wAlbB}}}{{2^{ - RPS17}}}$. Samples from isofemale line and tissue experiments were as follows; a total volume of 10 μL per reaction, each containing: 5 μL of 2x PerfeCTa SYBR Green SuperMix (Quantabio), 0.2 μL of each forward and reverse primers (10 μM), 2.6 μL of nuclease-free water, and 8uL of template DNA. The qPCR temperature profile for both experiments included denaturation at 95 °C for 5 min, 45 cycles of 95 °C for 10 s, 60 °C for 15 s and extension at 72 °C for 10 s, followed by a melt curve analysis. All samples were run once unless the melt curves suggested a failure in which case the sample would be rerun. If good melt curves were not obtained (rarely), the data were discarded. Rather than focus on technical replicates, where we tend to see very little variation, we focused our experimental efforts on biological replicates.

### Statistical analysis

Statistical analysis for the artificial selection study was performed in SPSS Statistics for Windows (IBM, Version 24.0). Density values were log10-transformed to reduce skewness. Statistical analysis for the isofemale line and tissue correlation experiment was performed in GraphPad Prism version 9.1.0 for Windows, GraphPad Software, San Diego, California USA. Data were checked for normality before performing analysis and transformed by log + 1 when necessary. All relative densities when depicted in scatter plots were plotted on a log axis. Fitted regression lines, although linear, can therefore appear curved. All posthoc comparisons were multiple test corrected using Tukey’s method.

## Results

### Artificial selection

**Fig. 1:**
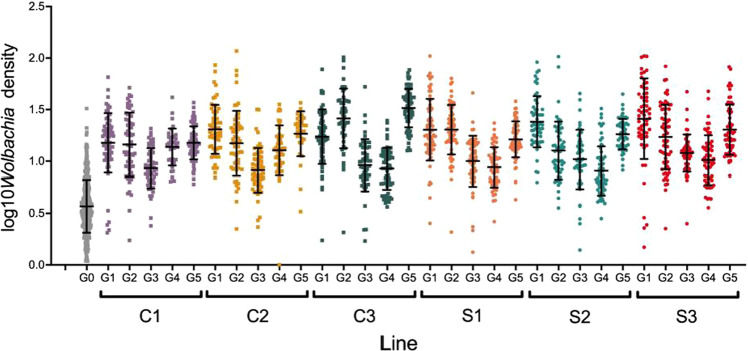
Relative *w*Mel *Wolbachia* density for parental (G0), control (C1-3) and artificially selected (S1-3) lines at each generation (G1-5). Density is expressed as the number of *Wolbachia tm513* gene copies normalized to the number of *rps17* gene copies. Error bars represent ±1 SD.

To determine whether artificial selection could be used to increase relative *Wolbachia* densities, *Ae. aegypti* mosquitoes were exposed to a selection regime for five generations. *Wolbachia* density was modeled using a mixed-effect model with generation and treatment as a fixed factor and line as a random factor nested within treatment. While treatment alone was not significant, there was a significant effect of generation (*F* = 17.39, df = 4, *p* = 0.001) and line (*F* = 4.19, df = 2, *p* = 0.015), typically densities were highest at generation 1, reached a minimum at generations 3–4, and then began climbing during generation 5 (Fig. 1). We also saw significant interactions between line and generation (*F* = 4.86, df = 8, *p* < 0.0001) and treatment and generation (*F* = 10.42, df = 4, *p* < 0.0001) (Fig. 1). Both treatments displayed the parabolic trend described above, but the control lines decreased in *Wolbachia* densities faster than our artificially selected lines. The control lines reached a minimum at generation 3. Of note is the increase in *Wolbachia* density across the board from the base population in all treatments and control lines. All samples had to be tested each generation (not blocked) because the relative densities were used specifically to select females to use for subsequent round of selection. We cannot, therefore, rule out that these shifts through time were due to differences between PCR runs. Regardless of these patterns, the selection regime did not increase density in the selected lines compared to controls. Repeating the statistical analysis with only generation 5 (endpoint) data also revealed no significant difference between selection and control-treated mosquitoes (*F* = 0.325, df = 1, *p* = 0.60), but there was a significant difference between lines (*F* = 28.40, df = 4, *p* < 0.0001). Control line 3 had the highest densities, while control line 1 had the lowest. In summary, we could not significantly shift *Wolbachia* densities based on whole-body estimates via artificial selection.

### Isofemale lines

**Fig. 2:**
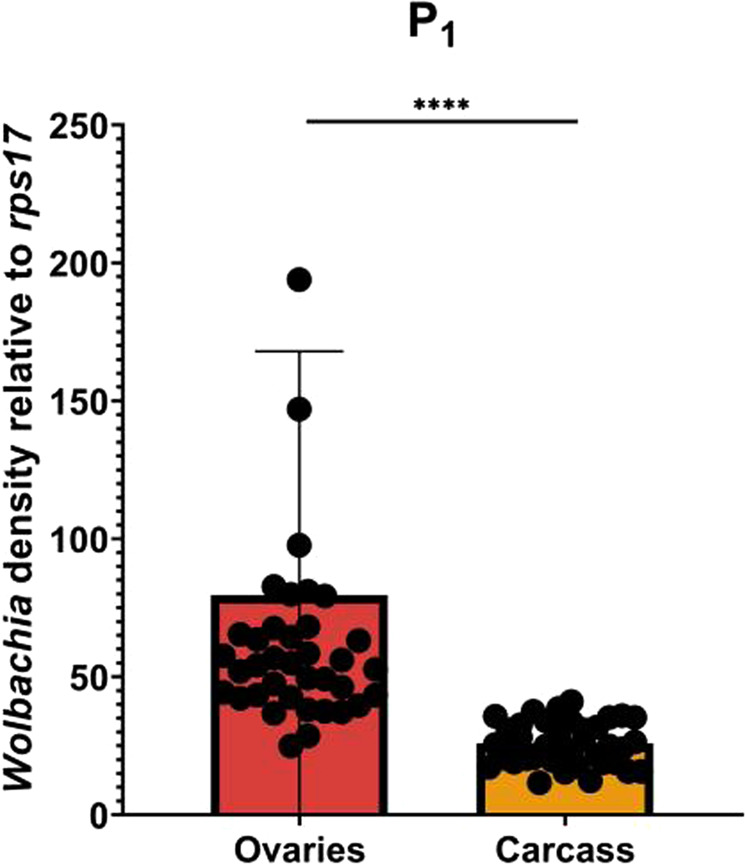
Relative *w*AlbB *Wolbachia* densities (*ankyrin repeat domain* to *rps17*) in the ovaries and the carcass of *Ae. aegypti* in the parental generation (P_1_). *n* = 39, *P* < 0.0001. Bars indicate tissue means ± SE; ****P ≤ 0.0001.

**Fig. 3:**
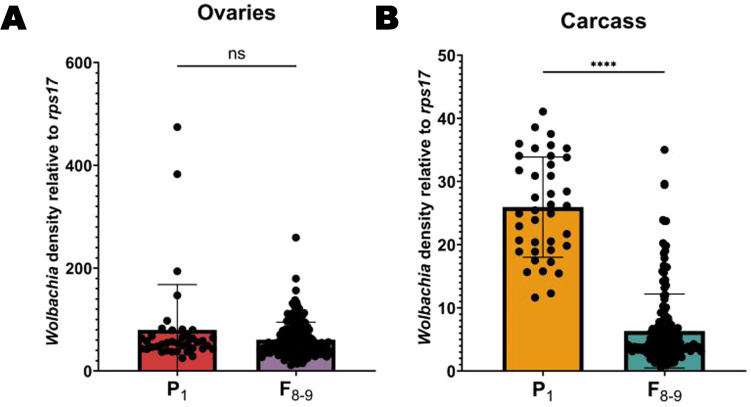
*Relative w*AlbB *Wolbachia* densities (*ankyrin repeat domain* to *rps17*) in the parental generation (P_1_) versus generations 8–9 (F_8-9_). **A** *Wolbachia* densities in the ovaries of P_1_ versus the ovaries of F_8–9_ in *Ae. aegypti*. **B** *Wolbachia* densities in the carcass of P_1_ versus the carcass of F_8–9_ in *Ae. aegypti*. For A and B, P_1_ *n* = 39 and F_8–9_ *n* = 170. Bars indicate tissue means ± SE; ns not significant; ****P ≤ 0.0001.

**Fig. 4:**
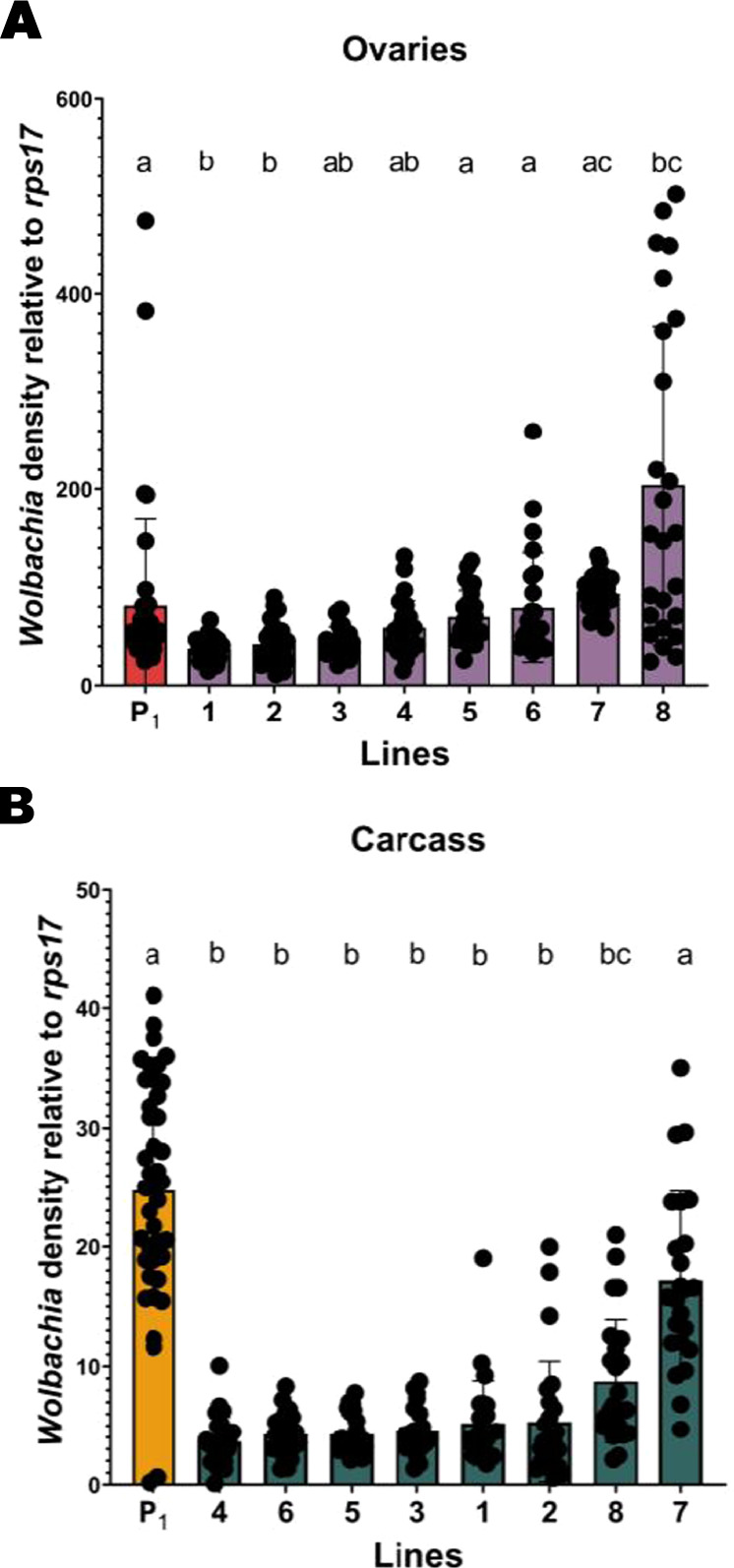
Relative *w*AlbB *Wolbachia* densities (*ankyrin repeat domain* to *rps17*) in each family line at generations 8–9 (families 1–8) versus the parental generation (P_1_). **A** *Wolbachia* densities in the ovaries. **B** *Wolbachia* densities in the carcass. Different letters indicate significant differences between families based on Tukey’s test at *P* ≤ 0.05. P_1_ has *n* = 41 individuals and families 1–8 have *n* = 24–25 individuals.

**Fig. 5:**
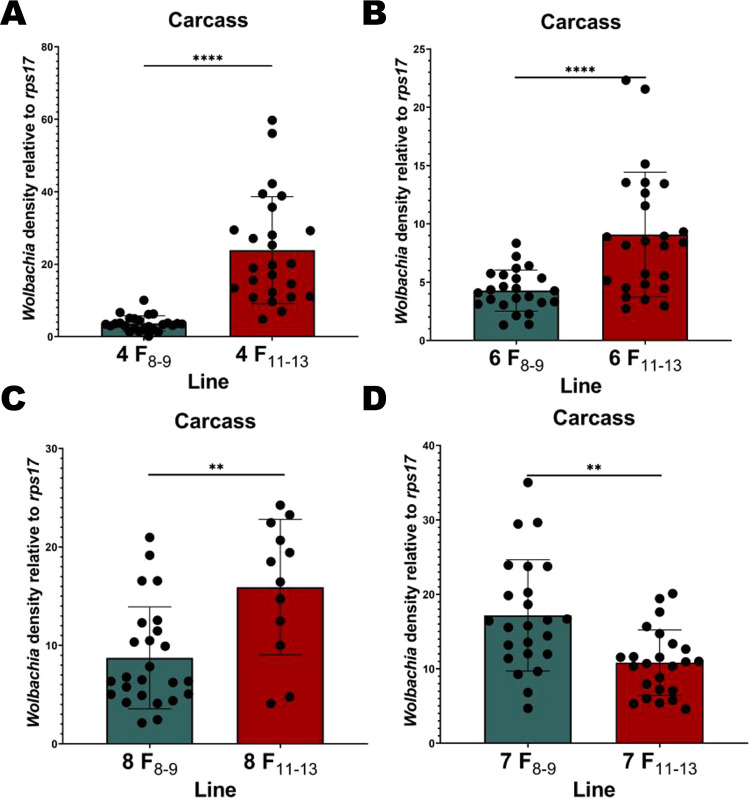
Relative *w*AlbB *Wolbachia* densities (*ankyrin repeat domain* to *rps17*) in the carcass for the lowest two (4, 6) and highest two (8, 7) family lines at generations 8–9 (F_8–9_) compared to their densities at generations 11–13 (F_11–13_). **A** *Wolbachia* densities for the line with lowest carcass density at F_8–9_ versus F_11–13_. **B** *Wolbachia* densities for the line with second-lowest carcass density at F_8–9_ versus F_11–13_. **C** *Wolbachia* densities for the line with highest carcass density at F_8–9_ versus F_11–13_. **D** *Wolbachia* densities for the line with second-highest carcass density at F_8–9_ versus F_11–13_. For **A** and **B** *n* = ~25 individuals. In **C**, 8 F_8–9_ *n* = 25 and at F_11–13_ *n* = 12. In **D**, *n* = 24 individuals. Bars indicate tissue means ± SE; **P ≤ 0.001; ****P ≤ 0.0001.

**Fig. 6:**
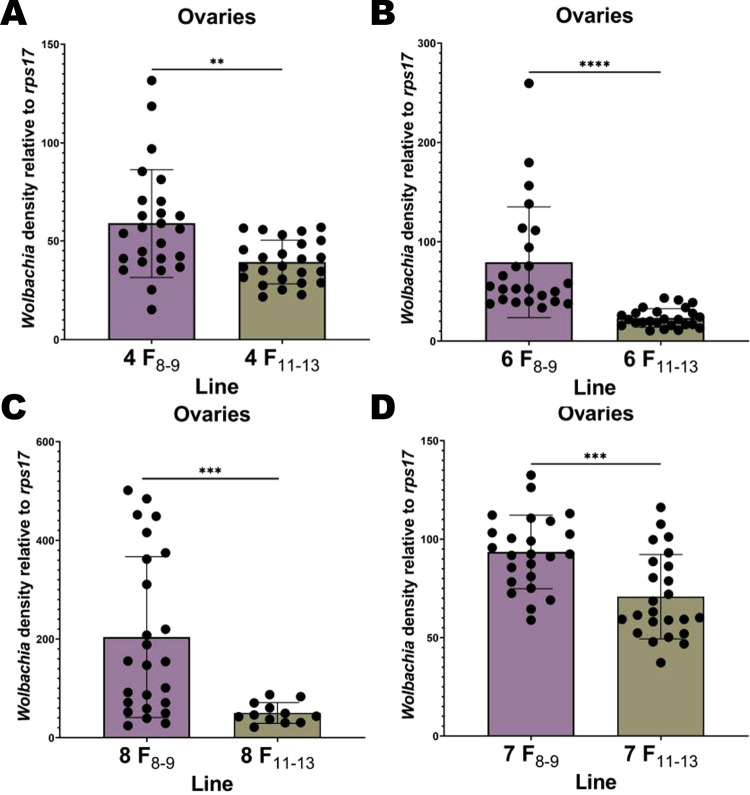
*w*AlbB Relative *Wolbachia* densities (*ankyrin repeat domain* to *rps17*) in the ovaries for family lines densities at generations 8–9 (F_8–9_) compared to their densities at generations 11–13 (F_11–13_). **A** *Wolbachia* densities for line 4 at F_8–9_ versus F_11–13_. **B** *Wolbachia* densities for line 6 at F_8–9_ versus F_11–13_. **C** *Wolbachia* densities for line 8 at F_8–9_ versus F_11–13_. **D** *Wolbachia* densities for line 7 at F_8–9_ versus F_11–13_. In **A** *n* = 25. In **B**, 6 F_8–9_ *n* = 24 and at F_11–13_ *n* = 25. In **C**, 8 F_8–9_ *n* = 25 and at F_11–13_ *n* = 12. In **D**, *n* = 24 individuals. Bars indicate tissue means ± SE; **P ≤ 0.001; 0.001 < ***P < 0.0001; ****P ≤ 0.0001.

In the original P_1_ generation, we measured relative *Wolbachia* densities in ovaries and carcasses of isofemales 6 days post blood feeding and post egg collection (15–17 days of adulthood). Relative densities ranged from ~25 to ~195 and from ~0 to ~41 in the ovaries and carcass, respectively (Fig. 2). This equated to a mean 2.3-fold higher density in ovaries than in carcass (*P* < 0.0001) (Fig. 2). We reassessed ovary and carcass densities after an additional 8–9 generations of rearing in our 8 remaining maternal lineages that survived the breeding process. Averaging across lines, we saw no change in ovary densities (*P* = 0.073) (Fig. 3A) and a significant decrease in the carcass density (*P* < 0.0001) (Fig. 3B) compared to P_1_. When examining isofemale lines individually, we found that ovary densities relative to P_1_ decreased for lines 1 and 2 but increased for line 8 (Supplementary Table 1). When comparing individual maternal lines to each other at F_8–9_, we found that line 8 was higher than most lines (Fig. 4A, Supplementary Table 1). Lines 5, 6 and 7 were also higher than several other lines. For carcass densities, we found a decrease for all lines compared to P_1_ except for 7 (Fig. 4B, Supplementary Table 2). Between F_8–9_ lines, we found that line 7 was higher than most lines, and line 8 was higher than several others (Fig. 4B, Supplementary Table 2). At generations F_11–13_, we reassessed lines with the two lowest (lines 4 & 6) and highest (lines 7 & 8) densities as measured at F_8–9_. The two lowest lines did not remain low, rebounding to high densities, and the two highest changed in opposite directions. All ovary densities in these same 4 lines exhibited a decrease. In summary, we saw a decrease in relative carcass densities after 8–9 generations of breeding and created lines with distinct *Wolbachia* densities. However, we could not maintain lines at characteristic high and low densities over multiple generations suggesting that isofemale line creation is not an avenue to generate stable and distinct densities through time (Figs. 5 and 6).

### Within individual tissue correlation

**Fig. 7:**
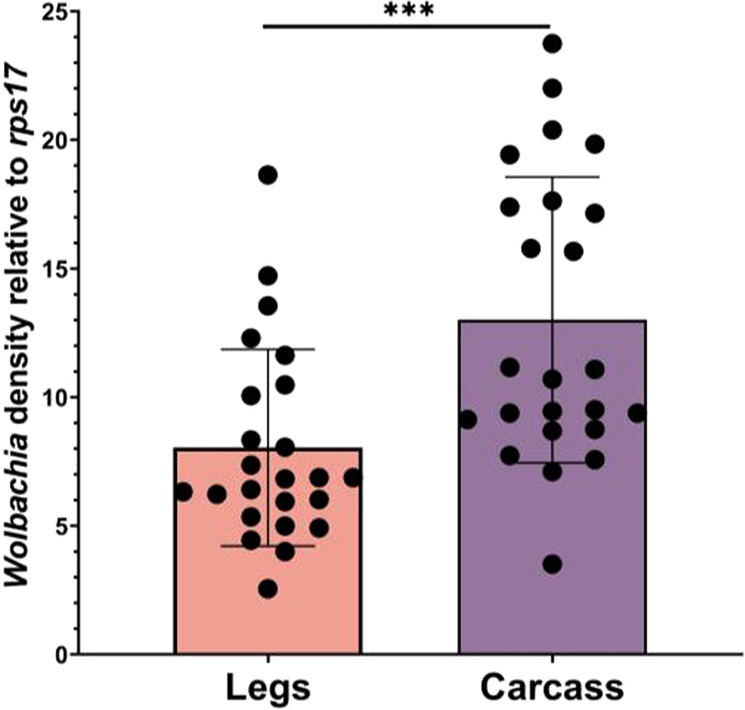
Relative *w*AlbB *Wolbachia* densities (*ankyrin repeat domain* to *rps17*) in the legs and the carcass of *Ae. aegypti*. *n* = 24, *P* < 0.0001. Bars indicate tissue means ± SE; 0.001 < ***P < 0.0001.

**Fig. 8:**
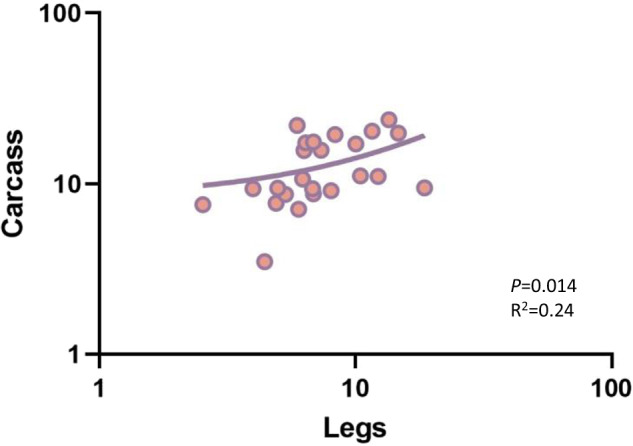
Relationship between relative *Wolbachia* densities (*ankyrin repeat domain* to *rps17*) in the legs and the carcass of *Ae. aegypti*. *n* = 24.

**Fig. 9:**
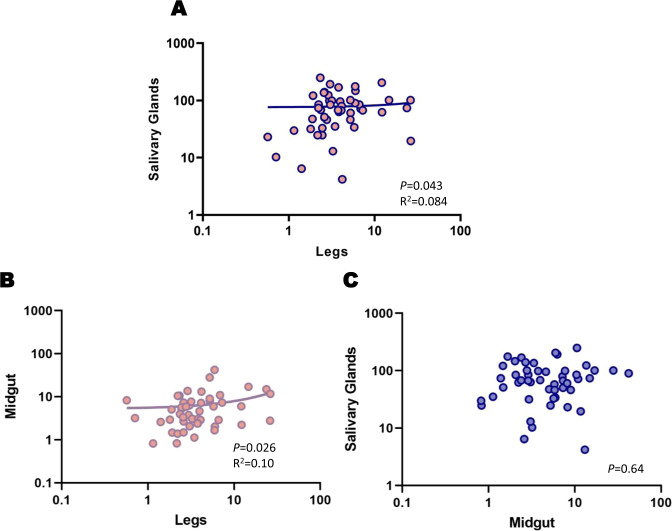
Relative *w*AlbB *Wolbachia* densities (*ankyrin repeat domain* to *rps17*) in the salivary glands, legs, and midgut of *Ae. aegypti*. **A** *Wolbachia* densities in the legs versus the salivary glands of *Ae. aegypti*. **B** *Wolbachia* densities in the legs versus the midgut of *Ae. aegypti*. **B** *Wolbachia* densities in the salivary glands versus the midgut of *Ae. aegypti*. Figures **A** and **B** have 24 individuals and Figure **C** has 25 individuals.

We measured relative *Wolbachia* densities in legs (pool of 6), carcass (body minus ovaries), salivary glands, and midgut ~6 days post blood feeding (15–17 days of adulthood) to see whether leg densities could be used to accurately predict relative *Wolbachia* densities in the remaining carcass and specific tissues in single individuals. Leg densities were significantly (*P* < 0.0001) lower (1.6-fold) than that of the total body densities (Fig. 7). Importantly, we found a positive correlation between leg and carcass densities (*P* = 0.014) (Fig. 8) with an R^2^ of 0.24, indicating some ability to use legs to predict tissue densities. We also found a correlation between salivary gland and leg densities (*P* = 0.043) and between midgut and leg densities (*P* = 0.026), but our R^2^ values, 0.084 (Fig. 9A), and 0.10 (Fig. 9B), respectively, are suggestive of poor predictive ability. We found no correlation between the salivary gland and midgut densities (*P* = 0.64) (Fig. 9C). For our leg and total carcass density dataset, we then binned the leg densities into categories of high or low based on the mid-point value of the total range in densities (16.09) and examined our accuracy in predicting high and low loads in the carcass, similarly binned based on the midpoint of their total density range (20.24). We found that we could accurately predict relative category in the carcass 70% of the time. By selectively focusing on legs at the extreme ends of the density range (top and bottom quartile), we could improve predictive accuracy up to 91% of the time. Taken together, our results suggest that leg density estimates can be used to accurately predict carcass densities, an approach that, while destructive, may be useful for studying the impact of *Wolbachia* on gene expression, viral loads, and metabolic phenotypes in the carcass.

## Discussion

We show that artificial selection and isofemale line creation are not effective strategies for isolating and generating genetically similar *Wolbachia* strain:mosquito infections in *Ae. aegypti*, that differ in their symbiont densities. Previously we studied relative *Wolbachia* densities in *Ae. aegypti* in the framework of a modified full sib design and showed that they varied by family (Terradas et al. 2017). Given maternal inheritance of *Wolbachia*, however, such patterns could not be labeled ‘heritable’, because the shared maternal environment could also be determining density. In keeping with our findings here, relative *Wolbachia* densities have previously exhibited poor predictability across generations in both *Ae. aegypti* (Mejia et al. 2022) and *Ae. albopictus* (Ahantarig et al. 2008). Regardless, having the ability to study the effect of variable *Wolbachia* densities would assist with dissecting the genetic basis of symbiont-induced traits, particularly given the inability to genetically modify *Wolbachia*. As a partial solution, we have found that within generation predictions, from legs to the remainder of the mosquito body, may allow sufficient predictability to bin mosquitoes a priori into the categories of low and high densities. Such an approach offers means to carry out various -omics studies on the mosquito body where the appropriate processing could not involve the collection of DNA for density assessment.

There are several possible explanations for why both artificial selection and isofemale line creation were unable to shift *Wolbachia* densities. The first is that our study design could suffer from low power. However, our estimate of power to detect differences between control and selected lines in the artificial selection given our strong sample sizes averaged ~0.85. Similarly, for the comparisons between the P_1_ and F_8-9_ or F_11-13_ generations for ovaries and carcass were ~1.0 given large differences between line means in our comparisons. Specifically, with respect to our artificial selection experiment, a decoupling of the whole-body density from that in the ovaries could also explain our result. A previous study in the mosquito *Culex quinquefasciatus* has shown just such a disconnect (Emerson and Glaser 2017). Second, with respect to both approaches, we may have lacked substantial genetic variation in either the *Wolbachia* or the host. Many studies have demonstrated that native hosts for *Wolbachia* have lower densities than artificially infected hosts (Bian et al. 2013; Miller et al. 2010; Osborne et al. 2012). Therefore, density is in part dictated by yet unknown genetic factors in the host that may include immunity (Rancès et al. 2012; Ye et al. 2013) or other aspects of mosquito physiologies. In *Wolbachia*, there is a positive correlation between gene copy numbers in the Octomom region of the *Wolbachia* genome in *D. melanogaster-*derived strains, and density demonstrating, that genetic factors in the bacterium also dictate loads (Chrostek et al. 2013; Chrostek and Teixeira 2015). Additionally, *w*AlbB relative density was found to be similar across the singly infected *Ae. aegypti* line and when found in co-infection in the same vector with the *w*Mel strain (Joubert et al. 2016), supporting our claim of *Wolbachia’s* genotype-based influences. Our selection and isofemale experiments were carried out with two different *Wolbachia* strain x mosquito population combinations that were optimized for high genetic variation in the vector but not the *Wolbachia*. The two populations we studied may still have lacked genetic variation for the specific trait of interest – vector control of *Wolbachia* loads. *Wolbachia*, in both lines will have much reduced genetic diversity, having initially been created through a single or handful of females that became infected via artificial transinfection (Walker et al. 2011; Xi et al. 2005). Additionally, we know from laboratory culturing and resequencing experiments that *Wolbachia* tends to evolve very slowly (Ross et al. 2022), likely due in part to the constraints of extreme bottlenecks at each generation in the insect.

A recent study in *D. melanogaster* infected with *w*Mel, showed that inbreeding caused relative *Wolbachia* densities in the whole body to reach a maximum in the host every 1–2 generations followed by an extremely low load in the next generation (Liu and Li 2021). We saw a similar pattern in both our artificial selection and isofemale experiments. This cycling could be explained by natural selection, interactions with environment, or PCR artifacts. One could imagine scenarios where factors that limit *Wolbachia* densities – such as insect immunity (Kambris et al. 2009; Ye et al. 2013), access to nutritional resources (Geoghegan et al. 2017; Kabouridis et al. 2000; Wu et al. 2004), or access to cellular niches (White et al. 2017), prevent *Wolbachia* loads from rising too high despite selection on the symbiont to maximize transmission. This could also be the case if the rising relative densities might be associated with fitness costs in the vector, as shown previously (Ant et al. 2018). Immune defense activities are themselves costly (Ahmed et al. 2002; Schwartz and Koella 2004), which may explain a balancing act for hosts and a cycling of *Wolbachia* loads, keeping *Wolbachia* levels in check within a reasonable range, while not over-reacting to them. Anecdotally, we frequently struggled to rear the isofemale lines with high *Wolbachia* loads, because they were less willing to blood feed and tended to produce smaller egg clutches. This mirrors what has been seen previously for the over replicating *w*MelPop strain both in flies (Min and Benzer 1997) and mosquitoes (McMeniman et al. 2009) that causes higher fitness costs, presumably due to greater *Wolbachia* loads. Our observation will need to remain speculative until future studies, as doing controlled fitness experiments was not possible with lines that were a struggle to maintain. Our goal was to fix vector genetic differences for relative densities across the isofemale lines, but we may have selected against that diversity instead. Previous studies in wasps (Mouton et al. 2003) and flies (Correa and Ballard 2012), also showed that variation in absolute and relative *Wolbachia* densities tended to decline with inbreeding, respectively. While we aimed to keep all environmental conditions (temperature, larval densities, etc.) consistent, they could explain how all lines in the artificial selection regime, including control lines, exhibited parallel cyclical changes in relative densities. Temperature can lead to increases or decreases in *Wolbachia* densities (Madhav et al. 2020; Mouton et al. 2003, 2007; Serbus et al. 2015). The *w*Mel strain currently used in field releases (Ulrich et al. 2016), is more sensitive than *w*AlbB (Ross et al. 2017) to temperature effects. Larval crowding and *ad libitum* food delivery have been shown specifically to limit *Wolbachia* densities, too (Dutton and Sinkins 2004; Wiwatanaratanabutr and Kittayapong 2009).

Our results do show a correlation between the relative density of *Wolbachia w*AlbB in the legs and the rest of the mosquito body (minus ovaries) that may be used with high accuracy to separate the remaining body into high and low relative density groups. *Wolbachia* loads in mosquitoes are known to vary heavily across mosquito tissues (Joubert et al. 2016) that could relate to effects, like the initial distribution of *Wolbachia* in the early embryo, the tissue-specific availability of appropriate cellular niches or resources (as above), variable activity of the vector immune response across tissues (Bonizzoni et al. 2012; Sim et al. 2012), or the differential replication of *Wolbachia* across particular cell/tissue types.

## Conclusion

Given the origins and history of *Wolbachia* in *Ae. aegypti* we expected the symbiont genome to contribute little genetic variation with respect to relative density determination. Despite working with outcrossed field populations of *Wolbachia*-infected mosquitoes, and evidence from the literature that both within and between species level variation (genetic diversity) (Kondo et al. 2005; McGraw et al. 2002; Mouton et al. 2007) can have effects on symbiont relative density, we saw little evidence for genetic or phenotypic variation within populations. These findings are in keeping with situations where *Wolbachia* numbers have been reassessed in field populations several years post release (Ahmad et al. 2021; Frentiu et al. 2014). When densities did shift in response to isofemale line creation or artificial selection, they tended to cycle within a narrow range. This suggests that either local tissue physiologies, interactions with the environment, or opposing forces of natural selection on the symbiont or vector are at play. These findings do not bode well for creating high and low-relative density lines for PB trait dissection or for field release. Continuing to explore naturally occurring distinct *Wolbachia* strains that vary in density genotype may be the only useful approach for field release (Gu et al. 2022). These findings do suggest that there are moderating forces acting on symbiont loads that may help to maintain stable densities in the field once strains are released (Ahmad et al. 2021; Frentiu et al. 2014). We have shown a destructive means for predicting high and low-density individuals from mosquito legs, that can be used for a range of -omics approaches that would not simultaneously allow *Wolbachia* density estimation.

## Supplementary information

Supp Table 1

Supp Table 2

## Data Availability

All raw data for the study can be found upon publication in figshare 10.6084/m9.figshare.19422296.

## References

[CR1] Ahantarig A, Trinachartvanit W, Kittayapong P (2008). Relative *Wolbachia* density of field-collected *Aedes albopictus* mosquitoes in Thailand. J Vector Ecol.

[CR2] Ahmad NA, Mancini MV, Ant TH, Martinez J, Kamarul GMR, Nazni WA (2021). *Wolbachia* strain *w*AlbB maintains high density and dengue inhibition following introduction into a field population of *Aedes aegypti*. Philos Trans R Soc Lond B Biol Sci.

[CR3] Ahmed AM, Baggott SL, Maingon R, Hurd H (2002). The costs of mounting an immune response are reflected in the reproductive fitness of the mosquito *Anopheles gambiae*. Oikos.

[CR4] Amuzu HE, McGraw EA (2016). *Wolbachia*-based dengue virus inhibition is not tissue-specific in *Aedes aegypti*. PLOS Negl Trop Dis.

[CR5] Ant TH, Herd CS, Geoghegan V, Hoffmann AA, Sinkins SP (2018). The *Wolbachia* strain *w*Au provides highly efficient virus transmission blocking in *Aedes aegypti*. PLOS Pathog.

[CR6] Axford JK, Ross PA, Yeap HL, Callahan AG, Hoffmann AA (2016). Fitness of *w*AlbB *Wolbachia* infection in *Aedes aegypti*: Parameter estimates in an outcrossed background and potential for population invasion. Am J Trop Med Hyg.

[CR7] Bian G, Xu Y, Lu P, Xie Y, Xi Z (2010). The endosymbiotic bacterium *Wolbachia* induces resistance to dengue virus in *Aedes aegypti*. PLOS Pathog.

[CR8] Bian G, Zhou G, Lu P, Xi Z (2013). Replacing a native *Wolbachia* with a novel strain results in an increase in endosymbiont load and resistance to dengue virus in a mosquito vector.. PLOS Negl Trop Dis.

[CR9] Bonizzoni M, Dunn WA, Campbell CL, Olson KE, Marinotti O, James AA (2012). Complex modulation of the *Aedes aegypti* transcriptome in response to dengue virus infection. PLOS One.

[CR10] Chouin-Carneiro T, Ant TH, Herd C, Louis F, Failloux AB, Sinkins SP (2020). *Wolbachia* strain *w*AlbA blocks Zika virus transmission in *Aedes aegypti*. Med Vet Entomol.

[CR11] Chrostek E, Marialva MSP, Esteves SS, Weinert LA, Martinez J, Jiggins FM (2013). *Wolbachia* variants induce differential protection to viruses in *Drosophila melanogaster*: a phenotypic and phylogenomic analysis.. PLOS Genet.

[CR12] Chrostek E, Teixeira L (2015). Mutualism breakdown by amplification of *Wolbachia* genes. PLOS Biol.

[CR13] Correa CC, Ballard JWO (2012). *Wolbachia* gonadal density in female and male *Drosophila* vary with laboratory adaptation and respond differently to physiological and environmental challenges. J Invertebr Pathol.

[CR14] Dutra HLC, Rocha MN, Dias FBS, Mansur SB, Caragata EP, Moreira LA (2016). *Wolbachia* blocks currently circulating Zika virus isolates in Brazilian *Aedes aegypti* mosquitoes. Cell Host Microbe.

[CR15] Dutton TJ, Sinkins SP (2004). Strain-specific quantification of *Wolbachia* density in *Aedes albopictus* and effects of larval rearing conditions. Insect Mol Biol.

[CR16] Emerson KJ, Glaser RL (2017). Cytonuclear epistasis controls the density of symbiont *Wolbachia pipientis* in nongonadal tissues of mosquito *Culex quinquefasciatus*. G3 Genes Genomes Genet.

[CR17] Flores HA, O’Neill SL (2018). Controlling vector-borne diseases by releasing modified mosquitoes. Nat Rev Microbiol.

[CR18] Ford SA, Albert I, Allen SL, Chenoweth SF, Jones M, Koh C (2020). Artificial selection finds new hypotheses for the mechanism of *Wolbachia*-mediated dengue blocking in mosquitoes.. Front Microbiol.

[CR19] Ford SA, Allen SL, Ohm JR, Sigle LT, Sebastian A, Albert I (2019). Selection on *Aedes aegypti* alters *Wolbachia*-mediated dengue virus blocking and fitness. Nat Microbiol.

[CR20] Fraser JE, O'Donnell TB, Duyvestyn JM, O'Neill SL, Simmons CP, Flores HA (2020). Novel phenotype of *Wolbachia* strain *w*Pip in *Aedes aegypti* challenges assumptions on mechanisms of *Wolbachia*-mediated dengue virus inhibition.. PLOS Pathog.

[CR21] Frentiu FD, Zakir T, Walker T, Popovici J, Pyke AT, van den Hurk A (2014). Limited dengue virus replication in field-collected *Aedes aegypti* mosquitoes infected with *Wolbachia*. PLOS Negl Trop Dis.

[CR22] Geoghegan V, Stainton K, Rainey SM, Ant TH, Dowle AA, Larson T (2017). Perturbed cholesterol and vesicular trafficking associated with dengue blocking in *Wolbachia*-infected *Aedes aegypti* cells. Nat Commun.

[CR23] Gu X, Ross PA, Rodriguez-Andres J, Robinson KL, Yang Q, Lau M-J (2022). A *w*Mel *Wolbachia* variant in *Aedes aegypti* from field‐collected *Drosophila melanogaster* with increased phenotypic stability under heat stress.. Environ Microbiol.

[CR24] Heaton NS, Perera R, Berger KL, Khadka S, LaCount DJ, Kuhn RJ (2010). Dengue virus nonstructural protein 3 redistributes fatty acid synthase to sites of viral replication and increases cellular fatty acid synthesis. Proc Natl Acad Sci USA.

[CR25] Hedges LM, Brownlie JC, O’Neill SL, Johnson KN (2008). *Wolbachia* and virus protection in insects. Science.

[CR26] Hien NT, Anh DD, Le NH, Yen NT, Phong TV, Nam VS (2022). Environmental factors influence the local establishment of *Wolbachia* in *Aedes aegypti* mosquitoes in two small communities in central Vietnam. Gates Open Res.

[CR27] Hoffmann AA, Iturbe-Ormaetxe I, Callahan AG, Phillips BL, Billington K, Axford JK (2014). Stability of the *w*Mel *Wolbachia* infection following invasion into *Aedes aegypti* populations.. PLOS Negl Trop Dis.

[CR28] Hoffmann AA, Montgomery BL, Popovici J, Iturbe-Ormaetxe I, Johnson PH, Muzzi F (2011). Successful establishment of *Wolbachia* in *Aedes* populations to suppress dengue transmission. Nature.

[CR30] Ikeda T, Ishikawa H, Sasaki T (2003). Infection density of *Wolbachia* and level of cytoplasmic incompatibility in the Mediterranean flour moth, *Ephestia kuehniella*. J Invertebr Pathol.

[CR31] Iturbe-Ormaetxe I, Walker T, O’Neill SL (2011). *Wolbachia* and the biological control of mosquito-borne disease. EMBO Rep.

[CR32] Joubert DA, Walker T, Carrington LB, De Bruyne JT, Kien DHT, Hoang NLT (2016). Establishment of a *Wolbachia* superinfection in *Aedes aegypti* mosquitoes as a potential approach for future resistance management. PLOS Pathog.

[CR33] Kabouridis PS, Janzen J, Magee AL, Ley SC (2000). Cholesterol depletion disrupts lipid rafts and modulates the activity of multiple signaling pathways in T lymphocytes. Eur J Immunol.

[CR34] Kambris Z, Cook PE, Phuc HK, Sinkins SP (2009). Immune activation by life-shortening *Wolbachia* and reduced filarial competence in mosquitoes. Science.

[CR35] Kondo N, Shimada M, Fukatsu T (2005). Infection density of *Wolbachia* endosymbiont affected by co-infection and host genotype. Biol Lett.

[CR36] Kraemer MUG, Reiner RC, Brady OJ, Messina JP, Gilbert M, Pigott DM (2019). Past and future spread of the arbovirus vectors *Aedes aegypti* and *Aedes albopictus*. Nat Microbiol.

[CR37] Lindsey ARI, Bhattacharya T, Newton ILG, Hardy RW (2018). Conflict in the intracellular lives of endosymbionts and viruses: A mechanistic look at *Wolbachia*-mediated pathogen-blocking.. Viruses.

[CR38] Liu XC, Li ZX (2021). Transmission of the *w*Mel *Wolbachia* strain is modulated by its titre and by immune genes in *Drosophila melanogaster* (*Wolbachia* density and transmission). J Invertebr Pathol.

[CR39] Livak KJ, Schmittgen TD (2001). Analysis of relative gene expression data using real-time quantitative PCR and the 2-ΔΔCT method. Methods.

[CR40] Madhav M, Brown G, Morgan JAT, Asgari S, McGraw EA, James P (2020). Transinfection of buffalo flies (*Haematobia irritans exigua*) with *Wolbachia* and effect on host biology.. Parasit Vectors.

[CR41] McGraw EA, Merritt DJ, Droller JN, O’Neill SL (2002). *Wolbachia* density and virulence attenuation after transfer into a novel host. Proc Natl Acad Sci USA.

[CR42] McMeniman CJ, Lane RV, Cass BN, Fong AWC, Sidhu M, Wang YF (2009). Stable introduction of a life-shortening *Wolbachia* infection into the mosquito *Aedes aegypti*. Science.

[CR43] Mejia AJ, Dutra HLC, Jones MJ, Perera R, McGraw EA (2022). Cross-tissue and generation predictability of relative *Wolbachia* densities in the mosquito *Aedes aegypti*. Parasites Vectors.

[CR44] Merle H, Donnio A, Jean-Charles A, Guyomarch J, Hage R, Najioullah F (2018). Ocular manifestations of emerging arboviruses: dengue fever, chikungunya, Zika virus, West Nile virus, and Yellow Fever. J Fr Ophtalmol.

[CR45] Miller WJ, Ehrman L, Schneider D (2010). Infectious speciation revisited: impact of symbiont-depletion on female fitness and mating behavior of *Drosophila paulistorum*. PLOS Pathog.

[CR46] Min KT, Benzer S (1997). *Wolbachia*, normally a symbiont of *Drosophila*, can be virulent, causing degeneration and early death. Proc Natl Acad Sci USA.

[CR47] Moreira LA, Iturbe-Ormaetxe I, Jeffery JA, Lu G, Pyke AT, Hedges LM (2009). A *Wolbachia* symbiont in *Aedes aegypti* limits infection with dengue, chikungunya, and *Plasmodium*. Cell.

[CR48] Mouton L, Henri H, Bouletreau M, Vavre F (2003). Strain-specific regulation of intracellular *Wolbachia* density in multiply infected insects. Mol Ecol.

[CR49] Mouton L, Henri H, Charif D, Boulétreau M, Vavre F (2007). Interaction between host genotype and environmental conditions affects bacterial density in *Wolbachia* symbiosis. Biol Lett.

[CR50] Nazni WA, Hoffmann AA, NoorAfizah A, Cheong YL, Mancini MV, Golding N (2019). Establishment of *Wolbachia* strain *w*AlbB in Malaysian populations of *Aedes aegypti* for dengue control. Curr Biol.

[CR51] Newton ILG, Savytskyy O, Sheehan KB (2015). *Wolbachia* utilize host actin for efficient maternal transmission in *Drosophila melanogaster*. PLOS Pathog.

[CR52] Osborne SE, Iturbe-Ormaetxe I, Brownlie JC, O’Neill SL, Johnson KN (2012). Antiviral protection and the importance of *Wolbachia* density and tissue tropism in *Drosophila simulans*. Appl Environ Microbiol.

[CR53] Pan X, Zhou G, Wu J, Bian G, Lu P, Raikhel AS (2012). *Wolbachia* induces reactive oxygen species (ROS)-dependent activation of the Toll pathway to control dengue virus in the mosquito *Aedes aegypti*. Proc Natl Acad Sci USA.

[CR54] Pinto SB, Riback TIS, Sylvestre G, Costa G, Peixoto J, Dias FBS (2021). Effectiveness of *Wolbachia*-infected mosquito deployments in reducing the incidence of dengue and other Aedes-borne diseases in Niterói, Brazil: a quasi-experimental study. PLOS Negl Trop Dis.

[CR55] Rainey SM, Martinez J, McFarlane M, Juneja P, Sarkies P, Lulla A (2016). *Wolbachia* blocks viral genome replication early in infection without a transcriptional response by the endosymbiont or host small RNA pathways. PLOS Pathog.

[CR56] Rancès E, Ye YH, Woolfit M, McGraw EA, O’Neill SL (2012). The relative importance of innate immune priming in *Wolbachia*-mediated dengue interference.. PLOS Pathog.

[CR57] Ross PA, Robinson KL, Yang Q, Callahan AG, Schmidt TL, Axford JK (2022). A decade of stability for *w*Mel *Wolbachia* in natural *Aedes aegypti* populations. PLOS Pathog.

[CR58] Ross PA, Wiwatanaratanabutr I, Axford JK, White VL, Endersby-Harshman NM, Hoffmann AA (2017). *Wolbachia* infections in *Aedes aegypti* differ markedly in their response to cyclical heat stress.. PLOS Pathog.

[CR59] Ryan PA, Turley AP, Wilson G, Hurst TP, Retzki K, Brown-Kenyon J (2020). Establishment of *w*Mel *Wolbachia* in *Aedes aegypti* mosquitoes and reduction of local dengue transmission in Cairns and surrounding locations in northern Queensland, Australia. Gates Open Res.

[CR60] Schwartz A, Koella JC (2004). The cost of immunity in the Yellow Fever mosquito, *Aedes aegypti* depends on immune activation. J Evol Biol.

[CR61] Serbus LR, White PM, Silva JP, Rabe A, Teixeira L, Albertson R (2015). The impact of host diet on *Wolbachia* titer in *Drosophila*. PLOS Pathog.

[CR62] Sim S, Ramirez JL, Dimopoulos G (2012). Dengue virus infection of the *Aedes aegypti* salivary gland and chemosensory apparatus induces genes that modulate infection and blood-feeding behavior.. PLOS Pathog.

[CR63] Souza-Neto JA, Powell JR, Bonizzoni M (2019). *Aedes aegypti* vector competence studies: a review. Infect Genet Evol.

[CR64] Teixeira L, Ferreira Á, Ashburner M (2008). The bacterial symbiont *Wolbachia* induces resistance to RNA viral infections in *Drosophila melanogaster*. PLOS Biol.

[CR65] Terradas G, Allen SL, Chenoweth SF, McGraw EA (2017). Family level variation in *Wolbachia*-mediated dengue virus blocking in *Aedes aegypti*. Parasites Vectors.

[CR66] Thannickal VJ, Fanburg BL (2000). Reactive oxygen species in cell signaling. Am J Physiol Lung Cell Mol Physiol.

[CR67] Ulrich JN, Beier JC, Devine GJ, Hugo LE (2016). Heat sensitivity of *w*Mel *Wolbachia* during *Aedes aegypti* development.. PLOS Negl Trop Dis.

[CR68] Utarini A, Indriani C, Ahmad RA, Tantowijoyo W, Arguni E, Ansari MR (2021). Efficacy of *Wolbachia*-infected mosquito deployments for the control of dengue. N Engl J Med.

[CR29] van den Hurk AF, Hall-Mendelin S, Pyke AT, Frentiu FD, McElroy K, Day A (2012). Impact of *Wolbachia* on infection with chikungunya and Yellow Fever Viruses in the mosquito vector *Aedes aegypti*. PLOS Negl Trop Dis.

[CR69] Walker T, Johnson PH, Moreira LA, Iturbe-Ormaetxe I, Frentiu FD, McMeniman CJ (2011). The *w*Mel *Wolbachia* strain blocks dengue and invades caged *Aedes aegypti* populations. Nature.

[CR70] Werren JH (1997). Biology of *Wolbachia*. Annu Rev Entomol Vol.

[CR71] Werren JH, Baldo L, Clark ME (2008). *Wolbachia*: Master manipulators of invertebrate biology. Nat Rev Microbiol.

[CR72] White PM, Serbus LR, Debec A, Codina A, Bray W, Guichet A (2017). Reliance of *Wolbachia* on high rates of host proteolysis revealed by a genome-wide RNAi screen of *Drosophila* cells. Genetics.

[CR73] Wiwatanaratanabutr I, Kittayapong P (2009). Effects of crowding and temperature on *Wolbachia* infection density among life cycle stages of *Aedes albopictus*. J Invertebr Pathol.

[CR74] Woolfit M, Iturbe-Ormaetxe I, Brownlie JC, Walker T, Riegler M, Seleznev A (2013). Genomic evolution of the pathogenic *Wolbachia* strain, *w*MelPop. Genome Biol Evol.

[CR75] Wu M, Sun LV, Vamathevan J, Riegler M, Deboy R, Brownlie JC (2004). Phylogenomics of the reproductive parasite *Wolbachia pipientis w*Mel: A streamlined genome overrun by mobile genetic elements. PLOS Biol.

[CR76] Xi Z, Dean JL, Khoo C, Dobson SL (2005). Generation of a novel *Wolbachia* infection in *Aedes albopictus* (Asian tiger mosquito) via embryonic microinjection. Insect Biochem Mol Biol.

[CR77] Xi Z, Khoo CCH, Dobson SL (2005). *Wolbachia* establishment and invasion in an *Aedes aegypti* laboratory population. Science.

[CR78] Xu J, Hopkins K, Sabin L, Yasunaga A, Subramanian H, Lamborn I (2013). ERK signaling couples nutrient status to antiviral defense in the insect gut. Proc Natl Acad Sci USA.

[CR79] Ye YH, Woolfit M, Rancès E, O’Neill SL, McGraw EA (2013). *Wolbachia*-associated bacterial protection in the mosquito *Aedes aegypti*. PLOS Negl Trop Dis.

[CR80] Zug R, Hammerstein P (2015). Bad guys turned nice? A critical assessment of *Wolbachia* mutualisms in arthropod hosts. Biol Rev Camb Philos Soc.

