# Supplementary material for: Attempts to use breeding approaches in Aedes aegypti to create lines with distinct and stable relative Wolbachia densities
Source: Heredity (Edinb). 2022 Jul 22;129(4):215–24. doi: 10.1038/s41437-022-00553-x (PMC9519544; doi:10.1038/s41437-022-00553-x)
Supplement: Supplementary file 1 — Supp Table 1 [file 41437_2022_553_MOESM1_ESM.pdf]

Tukey's multiple comparisons test	Summary	Adjusted P Value
P_1 vs. 1	**	0.0013
P_1 vs. 2	**	0.0057
P_1 vs. 8	****	<0.0001
1 vs. 5	**	0.0024
1 vs. 6	**	0.0016
1 vs. 7	****	<0.0001
1 vs. 8	****	<0.0001
2 vs. 5	**	0.0085
2 vs. 6	**	0.006
2 vs. 7	****	<0.0001
2 vs. 8	****	<0.0001
3 vs. 7	****	<0.0001
3 vs. 8	****	<0.0001
4 vs. 7	*	0.013
4 vs. 8	****	<0.0001
5 vs. 8	****	<0.0001
6 vs. 8	****	<0.0001
