# Supplementary material for: Attempts to use breeding approaches in Aedes aegypti to create lines with distinct and stable relative Wolbachia densities
Source: Heredity (Edinb). 2022 Jul 22;129(4):215–24. doi: 10.1038/s41437-022-00553-x (PMC9519544; doi:10.1038/s41437-022-00553-x)
Supplement: Supplementary file 2 — Supp Table 2 [file 41437_2022_553_MOESM2_ESM.pdf]

Tukey's multiple comparisons test	Summary	Adjusted P Value
P_1 vs. 4	****	<0.0001
P_1 vs. 6	****	<0.0001
P_1 vs. 5	****	<0.0001
P_1 vs. 3	****	<0.0001
P_1 vs. 1	****	<0.0001
P_1 vs. 2	****	<0.0001
P_1 vs. 8	****	<0.0001
4 vs. 7	****	<0.0001
4 vs. 8	***	0.0001
6 vs. 7	****	<0.0001
6 vs. 8	*	0.0347
5 vs. 7	****	<0.0001
3 vs. 7	****	<0.0001
1 vs. 7	****	<0.0001
2 vs. 7	****	<0.0001
2 vs. 8	*	0.012
7 vs. 8	**	0.0059
